# Rectal Tumour Staging with Endorectal Ultrasound: Is There Any Difference between Western and Eastern European Countries?

**DOI:** 10.1155/2016/8631381

**Published:** 2015-12-24

**Authors:** Anna Fábián, Renáta Bor, Klaudia Farkas, Anita Bálint, Ágnes Milassin, Mariann Rutka, László Tiszlavicz, Tibor Wittmann, Ferenc Nagy, Tamás Molnár, Zoltán Szepes

**Affiliations:** ^1^First Department of Internal Medicine, University of Szeged, Korányi Fasor 8-10, Szeged 6720, Hungary; ^2^Department of Pathology, University of Szeged, Állomás Utca 2, Szeged 6720, Hungary

## Abstract

*Background*. Rectal tumour management depends highly on locoregional extension. Rectal endoscopic ultrasound (ERUS) is a good alternative to computed tomography and magnetic resonance imaging. However, in Hungary only a small amount of rectal tumours is examined with ERUS. *Methods*. Our retrospective study (2006–2012) evaluates the diagnostic accuracy of ERUS and compares the results, the first data from Central Europe, with those from Western Europe. The effect of neoadjuvant therapy, rectal probe type, and investigator's experience were also assessed. *Results*. 311 of the 647 ERUS assessed locoregional extension. Histological comparison was available in 177 cases: 67 patients underwent surgery alone; 110 received neoadjuvant chemoradiotherapy (CRT); ERUS preceded CRT in 77 and followed it in 33 patients. T-staging was accurate in 72% of primarily operated patients. N-staging was less accurate (62%). CRT impaired staging accuracy (64% and 59% for T- and N-staging). Rigid probes were more accurate (79%). At least 30 examinations are needed to master the technique. *Conclusions*. The sensitivity of ERUS complies with the literature. ERUS is easy to learn and more accurate in early stages but unnecessary for restaging after CRT. Staging accuracy is similar in Western and Central Europe, although the number of examinations should be increased.

## 1. Background 

Rectal tumours require special diagnostic and therapeutic approach due to their location. This is partly attributable to the way they spread: they are characterised by transmural invasion; thus they infiltrate adjacent tissues in a relatively short time. The vicinity of the anal sphincter poses a problem, as the maintenance of continence should also be attempted besides the oncological principles. Due to their anatomy, rectal tumours may metastasise in several anatomical regions, and distant metastases might be formed in the liver and in the lungs via the vena cava system.

There are several therapeutic options available: endoscopic or surgical resection as well as irradiation and chemotherapy. A precise knowledge of the TNM-stage is crucial for choosing the adequate treatment option [1]. Hepatic and pulmonary metastases can be effectively identified with CT, but it is not appropriate for the evaluation of the tumour extension and the regional lymph nodes [2]. For these two complementary modalities are available: MRI and ERUS. Although MRI might be an ideal choice with its high accuracy, its limited availability prevents it from becoming a routine procedure in the clinical practice except for Western Europe [3–6]. Thus, in other parts of the world, including Hungary, ERUS remains the primary diagnostic tool for determining the depth invasion of the tumour [6–8]. This is due to the fact that ERUS is well tolerated by patients; it is simple, quick, easy to learn, accurate, and cost effective [9, 10]. The aim of this study was to assess the accuracy of ERUS in patients with rectal carcinoma in the routine clinical practice based on the data collected from a single centre in Hungary and to compare it with data from Western European countries.

## 2. Methods

The first endorectal ultrasound for tumour staging was performed on November 15, 2006, at our university center. In the present study, ERUS examinations aiming to determine the depth infiltration and lymph node metastases of rectal tumours were evaluated retrospectively. The data were obtained from the period between November 15, 2006, and December 31, 2012. Before the examinations, full-bowel preparation (polyethylene glycol-electrolyte solution or sodium picosulfate) was applied to empty the rectum. Endorectal ultrasounds were performed with a rigid rectoscope (Hitachi Aloka ASU-67 with mechanical radial (360°) transducer using 7.5–10 MHz frequency range) or a flexible echoendoscope. Two flexible probes were available: Olympus GF-UE 160 and Fujinon EG-530 UR (electronic radial (360°) probes, with 4 frequency options in the 5–10 and 5–12 MHz frequency range). ERUS examinations were carried out by two gastroenterologists who gained expertise in both endoscopic techniques and ultrasound diagnostics. In the initial period, several experts familiar with ultrasound diagnostics were present during the examinations, and the images were interpreted based on their common consensus. Later, the examiner interpreted the endosonographic image alone. Staging was based on the TNM classification. The endosonographically defined clinical stage was indicated with uT and uN. According to the clinical staging results, the tumorous lesion was removed surgically or endoscopically, or neoadjuvant therapy was first administered, according to the applicable oncological protocols. Besides the ERUS for initial staging, a second one was carried out on some of the patients who received neoadjuvant treatment, aiming to determine the current stage before surgery, to estimate downstaging, if there had been any. The final stage was determined after the pathological procession of the surgical specimens (pT, pN and ypT, ypN in case of patients who received neoadjuvant treatment). The required data were collected from MedSolution patient recording system. Only those patients were involved in our analysis whose histopathological results with the final tumour stage were available. Patients were divided into three groups depending on the neoadjuvant treatment. Patients in the first group underwent surgical intervention without previous oncological treatment. ERUS was performed after chemoradiotherapy on patients of the second group. In the third group, ERUS was performed first, but CRT was also necessary before surgery, because of the advanced stage of the disease. In the latter case, the later date of the histopathological findings, as well as the effect of CRT on staging, had also been taken into consideration in the evaluation of the accuracy. The accuracy of endorectal ultrasonography was evaluated by comparing uT, uN and yuT, yuN stages with the final pT, pN and ypT, ypN stages. The measure of correspondence was determined and was also characterised by Cohen's kappa coefficient. The overstaging and understaging rates were investigated as well. The sensitivity, specificity, positive predictive value (PPV), and negative predictive value (NPV) of ERUS were calculated for each tumour stage. Evaluating the N-staging accuracy, the ability of ERUS to recognise metastatic lymph nodes was investigated; therefore no difference was made between N1- and N2-stages. The operator-dependency of ERUS was also investigated as well as the extent to which the experience of the endosonographer (learning curve) affects the accuracy. The learning curve was determined on the group that did not receive neoadjuvant treatment. The correctness of the endosonographic diagnoses from a single examiner was also evaluated in correlation with the number of examinations performed. Our results were compared to the largest multicentre, prospective, countrywide, and real-life study conducted by Marusch et al. in Germany [11], as a representative of the staging accuracy of ERUS in Western Europe.

## 3. Results 

In the six-year study period, a total of 647 endorectal ultrasounds were performed. 311 of the examinations aimed to determine the locoregional extension of the tumour. 30 examinations failed due to inaccessible lesions (significant narrowing of the lumen or lesion located above the distance accessible with the probe), probe failure, or inadequate bowel preparation. Histopathological results with the final tumour stage were available in only 177 cases. In the other cases, the surgery and pathological procession was performed in another institution, and only the staging with ERUS was performed in our institution. 67 of the 177 patients underwent surgery without previous chemoirradiation within an average interval of 24 days after the endosonographical staging (Group I); the other 110 patients received oncological treatment prior to the surgery: ERUS was performed before the neoadjuvant treatment in 77 patients (Group III) and after that in 33 patients (Group II).

### 3.1. Accuracy of T-Staging

In terms of the T-staging accuracy, a significant difference was noted between the three groups. The correspondence was highest (72%) in the group that did not receive CRT, with Cohen's kappa coefficient of 0.482, indicative of a moderate correspondence. 11 cases (16%) were overestimated and 8 were underestimated (12%) (Table 1). In this patient group, the pathological examination of the resected tissue revealed T3 stage in 12 patients; thus primary oncological treatment would have been necessary according to the current therapeutic protocols. ERUS reported uT3 stage in 7 of these cases and uT2N1 in one of the cases; therefore understaging led to inappropriate treatment in only 4 patients. It should be noted, however, that in these four cases the time interval between the endosonography and the surgery was longer (an average of 38 days).

**Figure 1:**
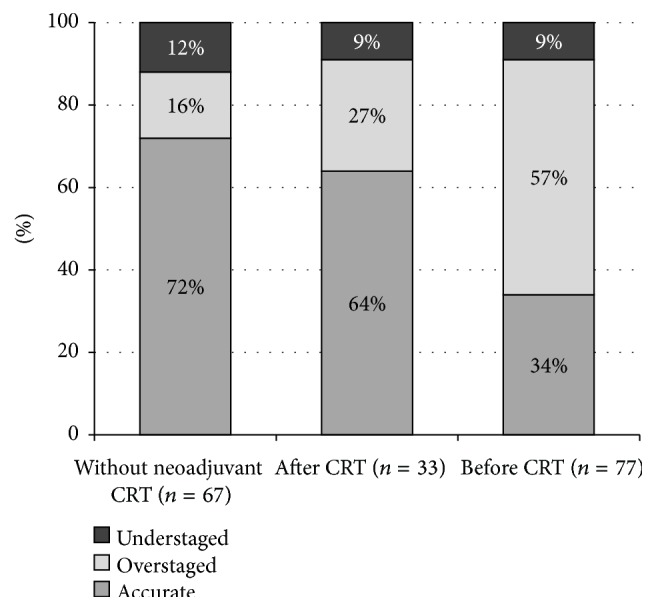
Accuracy of T-staging in each patient group.

The accuracy rate of ERUS was lower after neoadjuvant treatment (64%). In this group, overstaging was more frequent (27%) and 3 cases were understaged (9%). ERUS before CRT complied with the histopathological results in only 34% of the cases, accompanied by Cohen's kappa coefficient of 0.019 indicating poor correspondence. The overstaging rate was prominently high in this group (57%) (Figure 1).

In the majority of patients who did not receive CRT, early stage tumours were detected (the histopathological examination revealed pT1 in 61%, pT2 in 16%, and pT3 in 18% of the cases). At least moderate correspondence could be observed for each tumour category; the correspondence was highest for T3 tumours (*κ* = 0.606). Three-quarters of pT1 and pT2 tumours were identified correctly with ERUS (with a sensitivity of 75% and 73%, resp.), but, in case of T3 tumours, the sensitivity was only 58%. Unlike the high positive predictive values for T1 and T3 tumours, only 42% of the endosonographically defined T2 tumours were proved to be T2. The majority of uT2 cases were overestimated, as ERUS reported T2 instead of T1 (Table 2).

In accordance with the current protocols, the majority of the patients who received neoadjuvant treatment had T3 tumours. In two cases, the pathological results showed complete regression; no residual tumour tissue was detectable in the resected tissue. None of these could be identified endosonographically; the lesions were overestimated. It has been proven that the ERUS results of patients who received oncological treatment shifted towards overstaging compared to those who underwent surgery as a primary intervention (27% and 57% of the lesions were overstaged after and before the neoadjuvant treatment, resp.) (Figure 1).

After neoadjuvant CRT, the level of correspondence was lower for all of the T1-T3 stages. Correspondence was highest (*κ* = 0.525) for T3 tumours, 70% of the lesions described as yuT3 proved to be actually ypT3, and the sensitivity was 82%. A lower level of correspondence was observed in less advanced lesions; in T1 tumours, the sensitivity was only 20% and the positive predictive value was 50% (Table 3).

### 3.2. Accuracy of N-Staging

**Figure 2:**
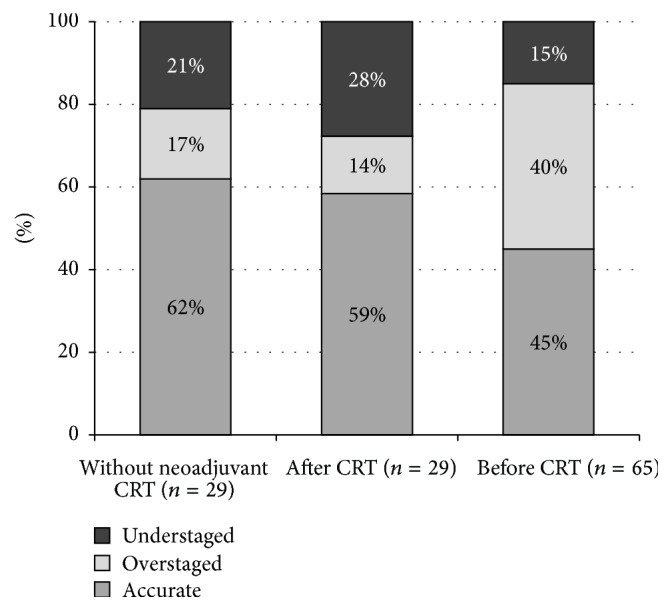
Accuracy of N-staging in each patient group.

Lymph node involvement was both reported with ERUS and mentioned in the histopathological findings in 123 patients. 29 of these patients underwent surgery as a primary treatment (Group I); the endosonographical staging preceded (Group III) and followed (Group II) CRT in 29 and 65 cases, respectively. In Groups I and II, the tumour stage seen with ERUS corresponded with the N-stage in the pathological results in 62% and 59% of the cases, respectively. This rate was significantly lower in Group III (45%). Understaging was more frequent in the former two categories (21% and 28%), while overstaging prevailed in the third one (40%) (Figure 2). It could also be noted that ERUS could more reliably recognise the absence of lymph node metastases than their presence (Table 4).

### 3.3. Learning Curve

**Figure 3:**
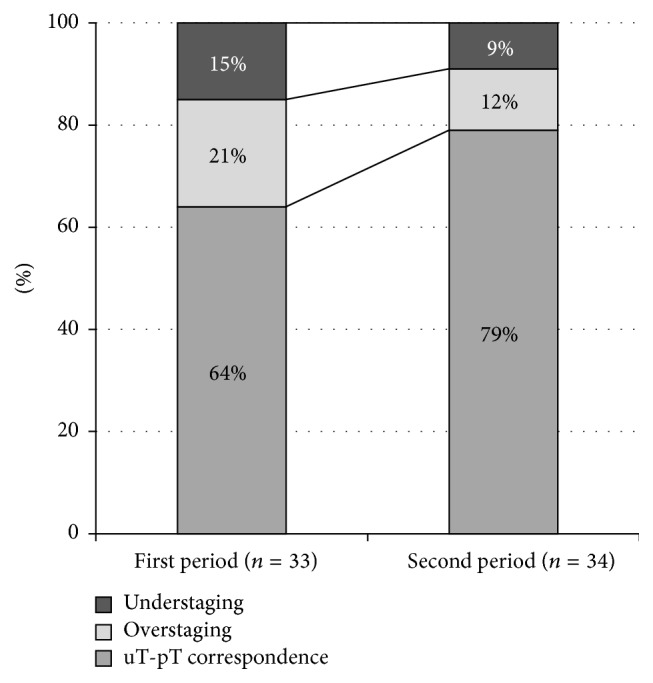
The accuracy of ERUS over time.

The time needed for gaining appropriate experience was investigated in the group that did not receive CRT; these 67 patients were divided into two groups; the 33 results of the initial period were compared to the subsequent 34 (Figure 3). The uT-pT correspondence was found to be significantly higher in the later period than in the initial one (*P* = 0.034). Furthermore, the understaging and overstaging rates both decreased. When taking into account only the cases from the later period, the sensitivity of ERUS reached 75% in all T-stages. All of the endoscopic T3 tumours were identified correctly (Table 5).

**Figure 4:**
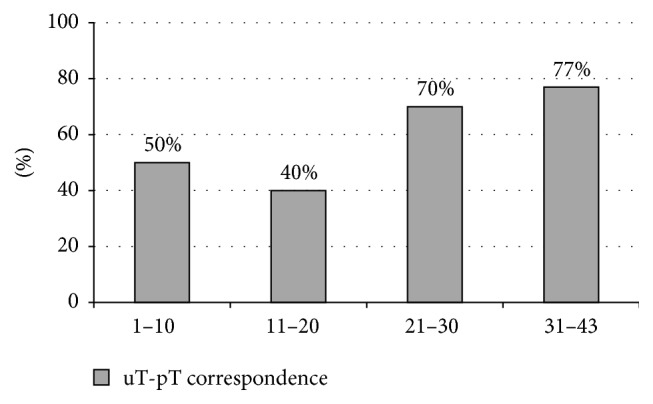
The performance of a single examiner after every 10 examinations.

The learning curve of one of the examiners was determined based on 43 ERUS examinations, by comparing the accuracy of the results divided into groups of 10 cases. The level of correspondence was found to be significantly higher after 30 examinations, suggesting a plateau phase or even a further increasing tendency (Figure 4).

Our case number was insufficient for determining the learning curve of the N-staging.

### 3.4. Accuracy of Rigid and Flexible Probes

**Figure 5:**
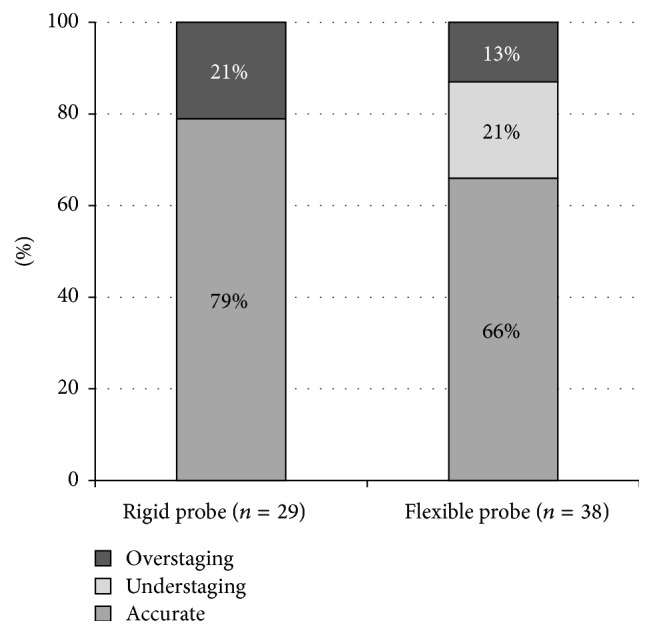
Accuracy of T-staging in case of flexible and rigid probes.

Rigid and flexible probes were compared based on their results in patients who did not receive CRT. 29 examinations were carried out with the rigid Aloka ASU-67 probe and 38 were carried out with the flexible Olympus GF-UE 160, Fujinon EG-530UT, and EG-530UR probes. The uT stage determined with the rigid probe showed a higher rate of correspondence with the final pT stage than the one defined with flexible probes (79% and 66%) (Figure 5). Inaccuracy of the rigid probe was exclusively due to overestimation, while underestimation could also be observed with the flexible devices; moreover, it was more frequent than overestimation. N-staging of the rigid probe could only be evaluated in six cases; the uN-pN stages were identical in five cases, and, in one case, the lymph node detected with ERUS did not prove to be metastatic. In case of the flexible devices, the results corresponded in 13 of the 23 cases; lymph node involvement was underestimated in six cases.

## 4. Discussion

The overall accuracy of ERUS in determining the depth invasion of the primary tumour (T stage) was found to be 72% in the patient group that did not receive CRT, with Cohen's kappa coefficient indicating moderate correspondence, which complies with the international data [11, 12]. According to a multicenter study performed in Germany, the overall accuracy of ERUS was determined as 73.1% for hospitals performing >30 ERUS/year. This rate was accomplished in our center as well. Overstaging was the most frequent mistake in all three patient groups (16%-27%-57%). The reason for this might be the so-called peritumoral inflammatory reaction, which cannot be distinguished endosonographically from the tumour itself [13, 14]. Understaging was mainly due to microscopic tumorous infiltration, which is impossible to detect with endosonography. It might as well occur in extensive tumours and when the upper part of the lesion is inaccessible for the probe. As the depth of invasion varies throughout the longitudinal extension of the tumour, an impairment in accuracy occurs when the tumour tissue cannot be examined as a whole [15, 16]. Differentiating between T1/T2 and T2/T3 tumours can raise further problems, as the penetration through the wall layers is often ambiguous; it might only be indicated by the irregularity of the surface between the layers. In case of extensive tumours, determining submucosal involvement might as well be difficult, as it can be easily mistaken for the widening of the muscular propria [15, 16]. Differentiating between T2/T3 tumours plays an important role in clinical decision-making, as the necessity of CRT depends on it. Out of the 67 cases five pT3 lesions were underestimated (three were reported as uT1 and two were reported as uT2); The overall clinical stage for one of the uT2 tumours was uT2N1. This means that, based solely on the endosonographical staging, 94% of the patients could receive adequate therapy, appropriate for the pathological stage. A significant variation in sensitivity was observed between T1-T2 and T3 stages in patients who underwent surgery without neoadjuvant CRT (75%-73% and 58%). It is ascertainable that while ERUS is a good diagnostic choice in case of early rectal malignancies, MRI is recommended for staging advanced lesions, due to its higher sensitivity [3, 17]. A significant difference was shown in terms of all the investigated parameters between the patient group that underwent surgery alone and the ones that received oncological treatment. This might be due to the effect of chemoirradiation on tissues: inflammation, fibrosis, and necrosis occurring as a consequence of the treatment can hardly be differentiated endosonographically from the tumorous tissue [18, 19]. The overstaging rate was 27–57% for Groups II and III, respectively. Taking into account the lower positive predictive value of the method and the fact that the level of yuT-ypT correspondence is only sufficient (*κ* = 0.390), it can be stated that ERUS itself is not appropriate for restaging after CRT. The yuT stage is not acceptable for evaluating the effectiveness of neoadjuvant therapy. ERUS performed prior to CRT reported a more advanced lesion than the final stage in a great percentage of the cases. Effective neoadjuvant treatment leads to a decrease in the tumour stage, which results in a discrepancy in the level of uT-pT correspondence and the overstaging rate compared to the patients who received no CRT [11].

The accuracy of N-staging was only 62%, and neither the sensitivity nor the positive predictive value of ERUS is acceptable. Therefore, it is inappropriate for the identification of metastatic lymph nodes. Currently, this is the greatest limiting factor of ERUS in rectal cancer staging. The method can only draw conclusions from the morphological features of lymph nodes to decide whether they are metastatic or not; however, there is no consensus about the staging criteria to be used [14, 20]. Most questions are being raised about the determination of the lymph node size that should be considered to be pathologic, as normal sized lymph nodes may also contain metastatic deposits, and, on the other hand, lymph node enlargement is not necessarily due to metastasis-formation. The facts that the evaluation of the perirectal fat is of limited availability on higher frequencies and that only lymph nodes adjacent to the rectum can be investigated with ERUS raise further problems [16].

Another limiting factor of ERUS is its operator-dependency. At the same time, this also means that in the hands of an experienced diagnostician it is a reliable method providing a great amount of information [21, 22]. According to our results, the learning curve is relatively short; after 30 examinations it is possible to evaluate the depth invasion of rectal cancers with confidence. Above this case load, the staging accuracy reached a significantly higher level (from 64% to 79%), which complies with the international statistics [11, 23]. Moreover, in the later period, after reaching the plateau phase of the learning curve, the sensitivity of ERUS for each tumour stage exceeded the results reported from a multicentre study from Germany (80%-83%-75% versus 58%-64%-71%) [11]. The reason for the better results in the initial period (first 10 examinations) after the introduction of ERUS to our institution might be the fact that several experts were present at the examinations and the endosonographical images were interpreted based on a common consensus. This could be a promising possibility for increasing the accuracy of ERUS in case of investigators without sufficient experience. Inevitably, regular practice is also crucial for high-level staging.

Flexible probes have several advantages over rigid ones: they are easier to manoeuvre with; due to their smaller diameter they are able to traverse a narrower lumen and to access higher locations than the rigid ones. Besides, a great advantage of flexible devices is the possibility of visual control, which is not available with rigid (“blind”) probes [15]. However, our results seem to support the fact that they stay behind the rigid probes in terms of both T- and N-staging. Thus, rigid probes are still favourable over flexible ones, due to their lower costs and higher accuracy [24].

## 5. Conclusions 

In the present study, ERUS was found to be of high accuracy in rectal tumour staging in accordance with the literature. No previous study has collected such extensive data on the accuracy of ERUS in the Central and Eastern European region. There is no significant difference between the accuracy of the modality in Central and Western European countries. After the relatively short learning curve, our results were even above the Western European standards, although they only represent the performance of a single centre not a countrywide analysis. ERUS is the method of choice for determining the depth invasion of the primary lesion in early malignancies, due to its simplicity, efficacy, low costs, and the fact that it is relatively well tolerated. After CRT, the inflammatory tissue reaction decreases its efficiency; thus it is not recommended for the evaluation of downstaging. However, the accuracy of ERUS might be similar in Central and Western Europe; one difference still remains: the extent of awareness of the modality and its availability. Although ERUS is becoming more and more widespread in Hungary, it is rarely part of the routine clinical practice. According to a questionnaire among 50 young surgeons in 2013, 51% of surgeons found ERUS the most accurate tool in terms of determining locoregional invasion, and only 33% of them uses it for staging; thus it is used in less than 10% of rectal cancers. Although some administration and organisation might be required from the attending physician, ERUS is utterly recommended for the adequate treatment of all patients with rectal carcinoma after the diagnosis and before oncological treatment.

## Figures and Tables

**Table 1:** Overall accuracy of T-staging throughout the whole study period (2006–2012).

	Group I (*n* = 67)	Group II (*n* = 33)	Group III (*n* = 77)
(y)uT-(y)pT correspondence	72%	64%	34%
Kappa coefficient	0.482	0.390	0.019
Overstaging	16%	27%	57%
Understaging	12%	9%	9%

**Table 2:** Accuracy of ERUS for each T stage without neoadjuvant therapy, throughout the whole study period (*n* = 67).

	uT1	uT2	uT3
uT-pT correspondence (kappa coefficient)	0.465	0.411	0.606
Sensitivity	75%	73%	58%
Specificity	74%	80%	96%
PPV	85%	42%	78%
NPV	61%	94%	91%

**Table 3:** Accuracy of ERUS for each T stage after neoadjuvant therapy (*n* = 33).

	yuT1	yuT2	yuT3
uT-pT correspondence (kappa coefficient)	0.218	0.415	0.525
Sensitivity	20%	67%	82%
Specificity	96%	83%	63%
PPV	50%	60%	70%
NPV	87%	87%	77%

**Table 4:** Accuracy of N-staging in each patient group.

	Group I (*n* = 29)	Group II (*n* = 29)	Group III (*n* = 65)
Sensitivity	14%	11%	50%
Specificity	77%	80%	42%
PPV	17%	20%	28%
NPV	74%	67%	66%

**Table 5:** Accuracy of ERUS for each T stage without neoadjuvant therapy, in the later study period after reaching a plateau in the learning curve (*n* = 34).

	uT1	uT2	uT3
uT-pT correspondence (kappa coefficient)	0.643	0.519	0.821
Sensitivity	80%	83%	75%
Specificity	86%	82%	100%
PPV	89%	50%	100%
NPV	75%	96%	93%

## Abbreviations

CRT: Chemoradiotherapy
CT: Computed tomography
ERUS: Endorectal ultrasound
MRI: Magnetic resonance imaging.
